# A Challenging Case of Recurrent Eosinophilic Peritonitis

**DOI:** 10.7759/cureus.9422

**Published:** 2020-07-27

**Authors:** Myra Nasir, Jasmin Hundal, Arish Noor, Juan Jose Chango Azanza, Jaimy Villavicencio

**Affiliations:** 1 Internal Medicine, University of Connecticut, Farmington, USA

**Keywords:** idiopathic eosinophilic peritonitis, eosinophilic gastroenteritis, ascites

## Abstract

Eosinophilic peritonitis is a rare presentation of eosinophilic gastroenteritis and is characterized by eosinophil-rich inflammation in any part of the gastrointestinal tract in the absence of secondary causes of eosinophilia. We report a case of a 48-year-old female who had recurrent hospital admissions due to abdominal pain and distension secondary to relapsing eosinophilic peritonitis.

## Introduction

Eosinophilic peritonitis (EP) is a rare presentation of eosinophilic gastroenteritis (EGE) [1]. Patients often present with abdominal distension, which can be accompanied by nausea, vomiting, diarrhea, and abdominal pain. The pathogenesis is poorly understood. We report the case of a 48-year-old female who had recurrent admissions for abdominal pain and distension and was found to have eosinophilic cholecystitis and EP.

## Case presentation

**Figure 1:**
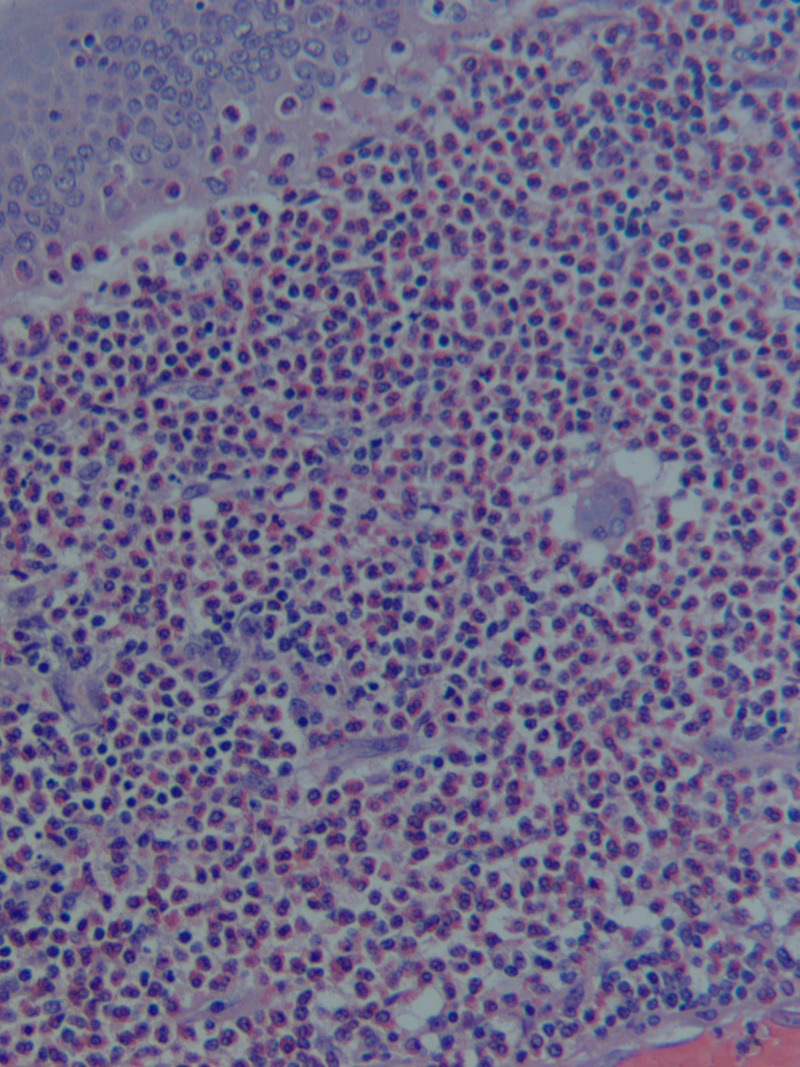
Inflammatory infiltrate consisting predominantly of eosinophils, suggesting eosinophilic cholecystitis

A 48-year-old female with a past medical history significant for asthma and bronchitis presented to the hospital in October 2018 with worsening abdominal pain associated with abdominal distension evolving over three weeks and diarrhea for three days. One month prior to this, she had undergone cholecystectomy, with tissue biopsy revealing eosinophilic cholecystitis (Figure 1). Her medications included furosemide 20 mg and pantoprazole 40 mg daily. She denied using any over-the-counter or herbal medications. Physical examination revealed a distended abdomen, diffusely tender to palpation. Laboratory tests showed white blood cell count of 17.7 x 10^3^/µL with an absolute eosinophil count of 1.2 K/µL (normal range: 0.0-0.5 K/µL) and albumin level of 2.50 g/dL. Liver enzymes and bilirubin levels were within the normal limits. A CT scan of the abdomen revealed large volume ascites with no evidence of small or large bowel dilation, abdominal mass, and cirrhosis. Doppler abdominal ultrasound revealed normal portal and hepatic venous flow. Diagnostic paracentesis revealed slightly cloudy, pale yellow fluid, 900 nucleated cells/µL with 75% eosinophils, 21% mononuclear cells, and 1.17 g/dL of albumin without evidence of malignancy. Ascitic fluid total protein and lactate dehydrogenase (LDH) levels were 4.3 g/dL and 64 U/L, respectively. Calculated serum ascites albumin gradient was 1.33 g/dL. Ascitic fluid bacterial culture and acid-fast bacillus (AFB) smear results showed no growth. Diagnosis of EP was made. MRI of the abdomen and MRCP (magnetic resonance cholangiopancreatography) were obtained, which were unremarkable. In addition to therapeutic paracentesis, she was treated with oral prednisone 40 mg/day, furosemide 20 mg/day, and spironolactone 100 mg/day, with improvement in her symptoms. She was discharged on a tapering schedule of prednisone.

The patient was readmitted to the hospital in February and again in March 2019 for abdominal pain and distension. She underwent paracentesis during both these admissions, with ascitic fluid study results showing elevated eosinophil count. There was no evidence of malignancy on cytology. She underwent extensive rheumatological, immunological, infectious, and genetic workups, which were largely unremarkable (Table 1). Upper endoscopy was performed, which appeared normal, and biopsies showed no evidence of eosinophilic esophagitis or gastritis. Colonoscopy findings were unremarkable for malignancy or inflammation. Random colonic biopsies were obtained, which showed normal colonic mucosa. CT scan of the chest, abdomen, and pelvis was remarkable for a cystic lesion in the right adnexa without abdominal or pelvic lymphadenopathy. Transthoracic echocardiogram revealed a normal ejection fraction of 55-65% with no other abnormalities. She was discharged on a tapering schedule of prednisone.

**Table 1:** Laboratory tests obtained for the work up of eosinophilic peritonitis. †ENA: RNP Ab, SM Ab, SSA Ab, SSB Ab, SCL 70 Ab, Jo 1 IgG Ab ‡Allergen, Northeast Region Profile: IgE for Oak Tree, Timothy grass, Blue grass, Ragweed, Lamb’s quarters, Cat epithelium, Dog dander, Cladosporium, Alternaria Tenuis, and Dermatophagoides Farinae. HBsAg, hepatitis B surface antigen; HBsAb, hepatitis B surface antibody; HIV, human immunodeficiency virus; Ab, antibodies; AFB acid-fast bacillus; Ig, immunoglobulin; ESR, erythrocyte sedimentation rate; CRP, C-reactive protein; ANA, anti-nuclear antibody; CH, hemolytic complement; MPO, myeloperoxidase; CCP, cyclic citrullinated peptide; ENA, extractable nuclear antibody; RF, rheumatoid factor; FISH, fluorescent in situ hybridization; RNP, ribonucleoprotein; SM, smooth muscle.

Laboratory test	Results	Normal Range
Infectious		
Hepatitis C antibody screen	Negative	Negative
HBsAg, HBsAb	Negative	Negative
HIV-1 and HIV-2 Ab	Negative	Negative
AFB (ascitic fluid)	No AFB isolated after 46 days	No AFB isolated
Strongyloides IgG	0.38 IV	≤0.99 IV
Aspergillus fumigatus IgE	<0.35 kU/L	<0.35 kU/L
Stool ova and parasite	Negative	Negative
Autoimmune		
ESR	32 mm/hr	0-20 mm/hr
CRP	10.1 mg/dL	0.0-0.8 mg/dL
ANA	Negative	Negative
CH 50	65 U/mL	42-95 U/mL
C3	149 mg/dL	83-177 mg/dL
C4	26 mg/dL	15-45 mg /dL
MPO Ab	4 units	≤20 units
Proteinase-3 Ab	3 units	≤20 units
CCP IgG	4 units	<20 units
ENA^†^	Negative	Negative
RF	<20 IU/mL	<30 IU/mL
Immunological		
IgG	1140 mg/dL	635-1741 mg/dL
IgA	249 mg/dL	66-433 mg/dL
IgE	23.4 IU/mL	<114.0 IU/mL
IgM	446 mg/dL	45-281mg/dL
Allergen, Northeast Region Profile^‡^	Negative	Negative
Genetic		
FISH CHIC 2 deletion	Normal	Normal
BCR/ABL	Normal	Normal

Out-patient follow-up in the hematology/oncology and gynecology clinics was arranged. The patient underwent bone marrow biopsy in April 2019, which revealed normal flow cytometry. Cytogenetics and FISH (fluorescence in situ hybridization) analysis for myeloproliferative neoplasm was normal. Serial transvaginal ultrasounds were performed, which showed a stable right adnexal mass, representing the confluence of a hydrosalpinx and normal ovary.

However, the patient was lost to follow-up in the hematology/oncology clinic and presented to the hospital in July 2019 with abdominal distension. She underwent paracentesis, with ascitic fluid studies remarkable for increased eosinophils. She was discharged on corticosteroids and has been maintained on 5 mg alternating with 12 mg of prednisone, which are continued indefinitely, 60 mg of furosemide, and 150 mg of spironolactone daily. Blood work obtained in November 2019 showed improvement in the absolute eosinophil count to 0.2 K/µL. She is closely followed up in the gastroenterology clinic and has reported no further complaints of abdominal distension to date.

## Discussion

EP has been reported in patients with EGE, hyper-eosinophilic syndrome (HES), and parasitic infections [2]. It is characterized by the presence of >100 eosinophils/µlL or eosinophils comprising of >10% of the non-erythrocyte count of the ascitic fluid [3]. HES is a myeloproliferative disorder which that was not seen in our patient. Parasitic infections were also ruled out. The most likely cause of EP in our patient is EGE, which consists of eosinophil-rich inflammation in any part of the gastrointestinal tract in the absence of secondary causes of eosinophilia [4]. In our patient, EGE manifested initially as eosinophilic cholecystitis.

EGE has been reported in all ages but is more common in children, young adults, and adults between the third and fifth decades of life [5]. Though it is more predominant in males, EP, which is a rare presentation of EGE, is more common in females [6]. EGE can be classified under mucosal, muscularis, and serosal forms, depending on the layer of the gastrointestinal wall where there is predominance of eosinophils [7]. The most common form is mucosal, which can present with abdominal pain, nausea, vomiting, and diarrhea. Serosal form is the least common and can present with eosinophilic ascites or EP. This can be accompanied by pleural effusion and rarely with ileus [6,8,9].

Diagnostic evaluation for EGE and EP includes exclusion of other causes of hypereosinophiliahypereosinophilia such as malignancy, parasitic infections, drug reactions, and other systemic/rheumatological diseases. Paracentesis with ascitic fluid analysis should be obtained, including cytology, cell count and differential, gram stain, culture, including AFB, glucose, protein, albumin, and LDH. It is important to rule out parasitic infections such as strongyloidiasisstrongyloidiosis before starting steroid therapy for EGE, in order to avoid disseminated infection. Endoscopic evaluation may show areas of inflammation, exudates, polyps, or stricture formation, and biopsies may show eosinophil-rich inflammation [4]. However, it is important to note that endoscopic appearance might be normal. Biopsies taken only in cases with abnormal endoscopic appearances can result in over more than 90% cases of EGE being missed [10]. Additionally, serosal forms of EGE can be missed if mucosal biopsies are taken. In such cases, full-thickness laparoscopic biopsies can be obtained [11].

Patients with EGE commonly report a history of allergies. Elimination of the allergen may result in symptom resolution. Since certain food allergies can contribute to EGE, diets eliminating certain foods such as milk, soy, eggs, wheat, peanuts, and shellfish have been recommended. These foods can be reintroduced one by one, and return of the symptoms can help identify the causative foods [12]. Steroids remain the mainstay of treatment. There are no established guidelines on the required duration of steroid use. A short course of steroid can be used, which should be repeated in cases of relapse. Some pPatients who experience refractory relapsing disease who may benefit from long-term low -dose steroid use [13]. Novel steroid-sparing therapies include anti-Ig-E and anti-IL-5 monoclonal antibodies such as omalizumab and mepolizumab, respectively [14,15].

## Conclusions

This case highlights EP/EGE as a diagnostic challenge, requiring a high index of suspicion and comprehensive work up. Serosal form of EGE, which can present with EP, can be missed if only mucosal gastrointestinal biopsies are taken. Steroids are the mainstay of treatment, and long-term treatment course may be required in patients with refractory relapsing disease.
